# Structural insights into the histidine-containing phospho­transfer protein and receiver domain of sensor histidine kinase suggest a complex model in the two-component regulatory system in *Pseudomonas aeruginosa*

**DOI:** 10.1107/S2052252520009665

**Published:** 2020-08-25

**Authors:** Shao-Kang Chen, Hong-Hsiang Guan, Pei-Hsun Wu, Li-Ting Lin, Meng-Chun Wu, Hwan-You Chang, Nai-Chi Chen, Chien-Chih Lin, Phimonphan Chuankhayan, Yen-Chieh Huang, Pei-Ju Lin, Chun-Jung Chen

**Affiliations:** a Department of Biotechnology and Bioindustry Sciences, National Cheng Kung University, Tainan City 701, Taiwan; b Life Science Group, Scientific Research Division, National Synchrotron Radiation Research Center, Hsinchu 30076, Taiwan; c Institute of Molecular Medicine, National Tsing Hua University, Hsinchu 30013, Taiwan; d Institute of Bioinformatics and Structural Biology, National Tsing Hua University, Hsinchu 30013, Taiwan; e Department of Physics, National Tsing Hua University, Hsinchu 30013, Taiwan; f Department of Biological Science and Technology, National Chiao Tung University, Hsinchu 30010, Taiwan

**Keywords:** two-component regulatory systems, sensor histidine kinase, histidine-containing phospho­transfer proteins, *Pseudomonas aeruginosa*

## Abstract

In *Pseudomonas aeruginosa*, an important opportunistic pathogen that causes numerous acute and chronic infections, the hybrid two-component system (TCS) regulates the swarming ability and biofilm formation with a multistep phospho­relay, and consists of hybrid-sensor histidine kinase (HK), histidine-containing phospho­transfer protein (Hpt) and response regulator (RR). In this work, two crystal structures of HptB and the receiver domain of HK PA1611 (PA1611REC) of *P. aeruginosa* have been determined in order to elucidate their interactions for the transfer of the phospho­ryl group. The structure of HptB folds into an elongated four-helix bundle – helices α2, α3, α4 and α5, covered by the short N-terminal helix α1. The imidazole side chain of the conserved active-site histidine residue His57, located near the middle of helix α3, protrudes from the bundle and is exposed to solvent. The structure of PA1611REC possesses a conventional (β/α)_5_ topology with five-stranded parallel β-sheets folded in the central region, surrounded by five α-helices. The divalent Mg^2+^ ion is located in the negatively charged active-site cleft and interacts with Asp522, Asp565 and Arg567. The HptB–PA1611REC complex is further modeled to analyze the binding surface and interactions between the two proteins. The model shows a shape complementarity between the convex surface of PA1611REC and the kidney-shaped HptB with fewer residues and a different network involved in interactions compared with other TCS complexes, such as SLN1-R1/YPD1 from *Saccharomyces cerevisiae* and AHK5_RD_/AHP1 from *Arabidopsis thaliana*. These structural results provide a better understanding of the TCS in *P. aeruginosa* and could potentially lead to the discovery of a new treatment for infection.

## Abstract 2

Two crystal structures of the histidine-containing phospho­transfer protein (HptB) and the receiver domain of the hybrid sensor histidine kinase PA1611 (PA1611REC) in the two-component regulatory system of *Pseudomonas aeruginosa* have been determined. With the modeled structure of the PA1611REC/HptB complex, the interactions and phospho­ryl group transfer between HptB and PA1611REC are elucidated.

## 1. Introduction   

In aiding the adaptation of cells to environmental changes, two-component regulatory systems (TCS) are widely distributed in prokaryotes, whereas few are identified in lower eukaryotic organisms and plants. The basic mechanism of the TCS signaling transduction is the transfer of a phospho­ryl group, which serves as a signaling molecule. The system is composed of a sensor histidine kinase (HK), which is auto­phospho­rylated at a conserved histidine residue of its cytoplasmic transmitter domain after sensing outside signals with the extracellular input domain; and a response regulator (RR), which accepts the phospho­ryl group at the conserved aspartate residue of the receiver domain (REC) from HK or a histidine-containing phospho­transfer protein (Hpt) (Stock *et al.*, 1989 ▸; 2000 ▸). The RR protein initiates the response by binding to a specific target protein, such as a transcriptional factor or DNA element (Bell *et al.*, 2010 ▸; Bauer *et al.*, 2013 ▸). The chemistry of the phospho­ramide and acyl­phosphate linkage is involved in this mechanism (Thomason & Kay, 2000 ▸). Biofilm formation and the implementation of drug resistance are typically regulated by TCS, which makes TCS an attractive target for research and drug intervention (Anjali *et al.*, 2019 ▸).

The sensor–regulator protein pairs in a TCS can be classified into three major types based on the organization of functional domains of HK: classic, unorthodox and hybrid systems (Rodrigue *et al.*, 2000 ▸; Lin *et al.*, 2006 ▸). In the classic system, a phosphate is transferred directly from phospho­rylated HK to RR (Skerker *et al.*, 2008 ▸; Capra *et al.*, 2010 ▸). In the unorthodox system, an extra receiver domain (REC) and a histidine-containing phospho­transfer (Hpt) domain are sequentially connected to the C-terminus of the HK transmitter domain. After the extracellular input domain of HK senses signals from the environment, the transmitter domain auto­phospho­rylates; the phosphate is then relayed to the receiver domain and the Hpt domain of HK. Subsequently, the Hpt domain transphospho­rylates the conserved aspartate residue at the receiver domain of RR (Posas *et al.*, 1996 ▸; Thomason & Kay, 2000 ▸; Zhao *et al.*, 2008 ▸; Kaserer *et al.*, 2010 ▸). The hybrid system is similar to the unorthodox system, but, instead of an Hpt domain attached to HK, an independent Hpt protein transfers the phospho­ryl group to the downstream RR (Rodrigue *et al.*, 2000 ▸).

*Pseudomonas aeruginosa* (*P. aeruginosa*) is a versatile Gram-negative bacterium grown in soil, coastal marine habitats, and on plant and animal tissues (Stover *et al.*, 2000 ▸). It is an elongated rod-shaped bacterium with only one-way flagellum mobility. Previous studies have shown that the metabolic versatility, intrinsic and acquired antibiotic resistance, biofilm formation and production of multiple virulence factors make *P. aeruginosa* a formidable pathogen causing numerous acute and chronic infections (Balasubramanian *et al.*, 2013 ▸). It accounts for 10–20% of all hospital-acquired infections and is the leading cause of chronic pulmonary infections and mortality in cystic fibrosis (CF) patients and burn victims (Stover *et al.*, 2000 ▸; National Nosocomial Infections Surveillance System, 2004 ▸; Lyczak *et al.*, 2002 ▸). In *P. aeruginosa*, the Hpt-mediated hybrid TCS serves as a basic stimulus–response coupling mechanism to decrease the environmental harm and to ensure survival by activating downstream responses, including antibiotic susceptibility, swarming activity and biofilm formation (Stock *et al.*, 2000 ▸; Wolanin *et al.*, 2002 ▸). Many TCS components have been described for their key roles during the infection processes of *P. aeruginosa* (Rodrigue *et al.*, 2000 ▸).

**Figure 1:**
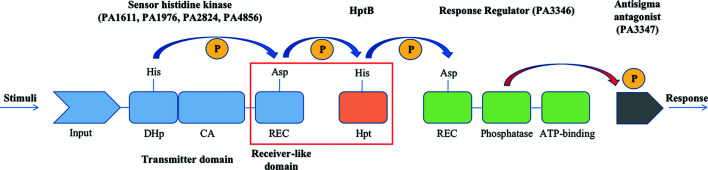
Schematic diagram of the phospho­relay in HptB-mediated hybrid TCS. The *P. aeruginosa* gene index numbers of the orphan sensors and the response regulator are listed in parentheses. Blue arrows indicate the phospho­ryl transfer in the pathway. The phospho­ryl group is presented with an encircled P. After activation with environmental stimuli, sensor histidine kinases (PA1611, PA1976, PA2824 and PA4856) auto­phospho­rylate and transfer the phospho­ryl group specifically to HptB, which in turn relays the signal to response regulator PA3346. The phospho­rylation regulates the Ser/Thr phosphatase activity of PA3346, resulting in increased phosphatase activity and de­phospho­rylation of the anti-sigma antagonist PA3347 (indicated with a red arrow). The phospho­rylation/de­phospho­rylation status might modulate the binding activity of PA3347 to other factor(s) and lead to the regulation of genes associated with swarming activity and biofilm formation.

The three orphan Hpt proteins in *P. aeruginosa* are HptA, HptB and HptC. HptB, the target protein in this work, is closely related to biofilm formation, which could increase drug resistance and protect the cell from pinocytosis by the host. According to previous studies, after the sensor proteins PA1611, PA1976 and PA2824 are auto­phospho­rylated, the phospho­ryl group is transferred to HptB and subsequently to the downstream RR protein PA3346 as illustrated in Fig. 1 ▸. The results showed that the HptB-mediated multistep phospho­relay plays a central role in the infection process of *P. aeruginosa* (Lin *et al.*, 2006 ▸; Hsu *et al.*, 2008 ▸). Moreover, the previous structures of Hpt proteins/domains in complex with receiver domains of HK or RR, such as SLN1-R1/YPD1 of yeast (PDB entry 2r25) and AHK5_RD_/AHP1 of *Arabidopsis thaliana* (PDB entry 4euk; Bauer *et al.*, 2013 ▸), have shown that the Hpt protein/domain would interact with the receiver domain. To elucidate the HptB-mediated TCS pathway of *P. aeruginosa* in depth, we report two crystal structures of HptB and the C-terminal receiver domain of HK PA1611 (PA1611REC). The structural details allow us to model the complex of HptB and PA1611REC, which reveals the possible binding conformation and the residues involved in the interaction between the two proteins. The result would further clarify the mechanism in the HptB-mediated signal transduction pathway of *P. aeruginosa* and potentially lead to the discovery of a new treatment for *P. aeruginosa* infection, such as novel small molecules which could potentially interfere with the interaction between HptB and the corresponding receiver domains.

## 2. Materials and methods   

### 2.1. Cloning and expression of target proteins   

Native HptB was cloned into pET23a (Novagen) using NdeI and XhoI restriction sites. For the mutant HptB, the only cysteine (C75) was mutated to alanine by polymerase chain reaction (PCR) overlap extension. The C-terminal fragment (a.a. 507–651) including the receiver domain of HK PA1611 (PA1611REC) was amplified by PCR and cloned into pET28a (Novagen) using NheI and EcoRI restriction sites with T4 DNA ligase (Invitrogen). The primers used are listed in Table 1 ▸. *E. coli* strain DH5α was used for the plasmid preparation and recombinant DNA manipulation.

After DNA sequencing confirmed the correctness of the insert, the expression constructs of both native HptB and PA1611REC were transformed into *E. coli* strain BL21(DE3) for the expression of recombinant proteins. The bacteria were cultured with shaking in Luria–Bertani media supplemented with specific antibiotics (100 µg ml^−1^) until the optical density at 600 nm (OD600) reached 0.6. Overexpression of the target protein was induced on adding iso­propyl-β-d-thio­galactoside (IPTG) to the final concentrations of 0.5 m*M* and 0.1 m*M* for native HptB and PA1611REC, respectively. The culture was grown overnight at 20°C with shaking and harvested using centrifugation at 8000*g* for 25 min at 4°C.

For the production of seleno­methio­nine-labeled (SeMet) mutant HptB, *E. coli* strain BL21-Gold (DE3) harboring the expression vector served as the host cell. The culture was grown in M9 medium (6 mg l^−1^ Na_2_HPO_4_, 3 mg l^−1^ KH_2_PO_4_, 0.5 mg l^−1^ NaCl, 1 mg l^−1^ NH_4_Cl, 1 m*M* MgSO_4_, 0.1 m*M* CaCl_2_, 1 mg l^−1^ thi­amine-HCl, 0.2% glucose) with l-lysine, l-threonine, l-phenyl­alanine (120 mg l^−1^), l-leucine, l-isoleucine, l-valine, l-seleno­methio­nine (60 mg l^−1^) and ampicillin (100 µg ml^−1^) until the optical density at 600 nm (OD600) reached 0.6. The following steps for the expression and purification of the SeMet-labeled protein were the same as the native HptB protein.

### 2.2. Purification of HptB protein   

The cell pellet from a 1 l culture was suspended in a lysis buffer (35 ml) consisting of Tris–HCl (50 m*M*, pH 8.0) and subjected to cell disruption by ultrasonication using a pulsation cycle 2 s on and 3 s off with a total duration of 20 min at 40% energy on ice. The soluble protein extract was collected by centrifugation at 12 000*g* for 30 min at 4°C and passed through an open column packed with resin (Q Sepharose Fast Flow, 60 ml, GE Healthcare Bio-Sciences AB, Uppsala, Sweden), which was equilibrated with binding buffer (90 ml) consisting of Tris–HCl (50 m*M*, pH 8.0). The column was washed with binding buffer (90 ml) containing (NH_4_)_2_SO_4_ (40 m*M*). The target protein was eluted with portions (90 ml) of binding buffer containing (NH_4_)_2_SO_4_ at stepwise-increasing concentrations of 60 and 100 m*M*. The eluted fractions were concentrated by the centricon of a 3 kDa filter unit (Amicon Ultra, Merck KGaA, Darmstadt, Germany) and then loaded onto a column (HiLoad 16/60 Superdex 75 PG, GE Healthcare Bio-Sciences AB) connected to a liquid chromatography (FPLC) system (ÄKTA, GE Healthcare) and equilibrated with a buffer composed of NaCl (300 m*M*) and Tris–HCl (20 m*M*, pH 8.0). The size-exclusion chromatography step yielded homogenous protein fractions.

### 2.3. Purification of PA1611REC protein   

The procedures of cell disruption and collection of the soluble protein extract were the same as those for the purification of HptB except for the lysis buffer, which was composed of NaCl (500 m*M*) and Tris–HCl (50 m*M*, pH 8.0). The extract was then passed through an open column packed with resin (10 ml Ni-Sepharose 6 Fast Flow, GE Healthcare Bio-Sciences AB, Uppsala, Sweden), which was equilibrated with a binding buffer (50 ml) consisting of NaCl (500 m*M*) and Tris–HCl (50 m*M*, pH 8.0). The column was washed with a binding buffer (50 ml) containing imidazole (20 m*M*). The target protein was eluted with portions (50 ml) of binding buffer containing imidazole at stepwise-increasing concentrations of 80, 100, 300 and 500 m*M*.

### 2.4. Crystallization   

After the eluted fractions were collected and verified with SDS–PAGE (15%) followed by staining (Coomassie Brilliant Blue R-250), the purified HptB protein was dialyzed against Tris–HCl (50 m*M*, pH 7.5) and concentrated to ∼8 mg ml^−1^ with a Centricon 3 kDa filter unit (Amicon Ultra, Merck KGaA, Darmstadt, Germany), whereas the purified PA1611REC protein was dialyzed against distilled water and concentrated to ∼10 mg ml^−1^ by the same method. Initial screening of crystallization conditions was performed by the hanging-drop vapor-diffusion method in 96-well plates with a liquid-handling robot (Mosquito, TTP Labtech). The hanging drops formed on mixing protein solution (0.1 µl) with reservoir solution (0.1 µl) and equilibrated against the reservoir solution (100 µl). To optimize the protein crystals, the hanging drops were made on mixing a protein solution (1 µl) with a reservoir solution (1 µl) and equilibrated against a reservoir solution (120 µl) in VDX48 plates (Hampton Research). All plates were placed in an incubator at 18°C.

Crystals formed under several conditions. The crystal of SeMet-labeled mutant HptB, grown in a reservoir solution containing PEG20000 [12%(*w*/*v*)] and MES monohydrate (0.1 *M*, pH 6.5), was picked for data collection. The structure of native HptB determined was from the crystals grown in a reservoir solution containing PEG6000 [10%(*w*/*v*)] and bicine/sodium hydroxide (100 m*M*, pH 9.0). For PA1611REC, the structure determined was from crystals grown in a reservoir solution containing PEG4000 [15%(*w*/*v*)], MgCl_2_ (0.2 *M*) and Tris–HCl (100 m*M*, pH 8.5).

### 2.5. Data collection and processing   

The crystals were transferred from the hanging drops into a cryoprotectant solution (1 µl) made from adding glycerol [20%(*v*/*v*)] into the reservoir solution for a few seconds. The crystals were then picked with synthetic nylon loops (Hampton Research, Aliso Viejo, CA, USA) and flash-cooled in liquid nitro­gen. The X-ray diffraction datasets were collected on beamlines TPS 05A, BL13B1 and BL15A1 at the National Synchrotron Radiation Research Center (NSRRC), Taiwan, and BL44XU and BL12B2 at SPring-8, Japan. For SeMet-labeled HptB, a multi-wavelength anomalous-dispersion (Se-MAD) experiment was performed. All datasets were indexed, integrated and scaled using the *HKL2000* program suite (Otwinowski & Minor, 1997 ▸). The data collection statistics are listed in Table 2 ▸.

### 2.6. Structure determination and refinement   

The positions of nine selenium atoms of three SeMet-labeled mutant HptB–C75A molecules in the asymmetric unit of space group *I*4_1_22 were automatically determined and used to calculate the multi-wavelength anomalous-diffraction phases with *SOLVE* (Terwilliger & Berendzen, 1999 ▸). The phases of wild-type HptB in space group *P*4_3_2_1_2 and PA1611REC in space group *P*3_1_21 were determined with the molecular replacement method using *Phaser MR* in the *CCP*4 program suite (Winn *et al.*, 2011 ▸), with the structures of *ab inito* determined mutant HptB–C75A and the receiver domain from yeast histidine kinase SLN1 (PDB entry 2r25; B-chain) as the search models, respectively. Structural refinement was performed using alternating rounds of model building with the program *Coot* (Emsley *et al.*, 2010 ▸) and restrained refinement with *Refmac5* in the *CCP4* program suite (Murshudov *et al.*, 2011 ▸) to improve the value of the *R* factor and *R*_free_. The refinement statistics are listed in Table 2 ▸. The secondary structure was assigned with the *DSSP* web server (Kabsch & Sander, 1983 ▸; Touw *et al.*, 2015 ▸), and the structure figures were prepared using *PyMOL* (http://www.pymol.org/).

### 2.7. Modeling of the HptB–PA1611REC complex   

To establish the complex model of HptB and PA1611REC, docking was performed using the crystal structures of two individual proteins with the *HADDOCK* web server (version 2.2; van Zundert *et al.*, 2016 ▸) in the easy interface mode. His57 of HptB and Asp565 of PA1611REC were considered the active residues because these two residues directly participate in the transfer of the phospho­ryl group according to previous studies, which confirmed this transfer between PA1611 and HptB (Lin *et al.*, 2006 ▸; Hsu *et al.*, 2008 ▸). The passive residues, which potentially make contact in the complex, were automatically defined in the easy interface mode that included all residues within a radius of 6.5 Å from the active residue and on the surface with relative surface accessibility of either a main chain or a side chain above 15% as determined with *NACCESS* (Hubbard & Thornton, 1993 ▸). The divalent metal ions (Mg^2+^) in the active-site cleft of PA1611REC were also applied to the *HADDOCK* web server. The complex models were clustered according to the pairwise backbone root-mean-square deviation (RMSD) at the interface; the clusters were sorted according to a *HADDOCK* score calculated from energies of van der Waals, electrostatic interaction, desolvation, restraint violation and buried surface area. The *HADDOCK* web server clustered 115 structures into eight clusters, which represents 57.5% of the water-refined models generated by *HADDOCK* for the HptB–PA1611REC complex with Mg^2+^. The cluster with the lowest *HADDOCK* score is the most likely solution; the first model in this cluster was chosen for the analysis of the interface with the *PDBePISA* web server (Krissinel & Henrick, 2007 ▸). The score of cluster 1 is −63.7 ± 2.6, whereas other clusters range from −48 to −7.6, indicating that the highest score is a unique solution.

## 3. Results   

### 3.1. Overall structure of HptB   

**Figure 2:**
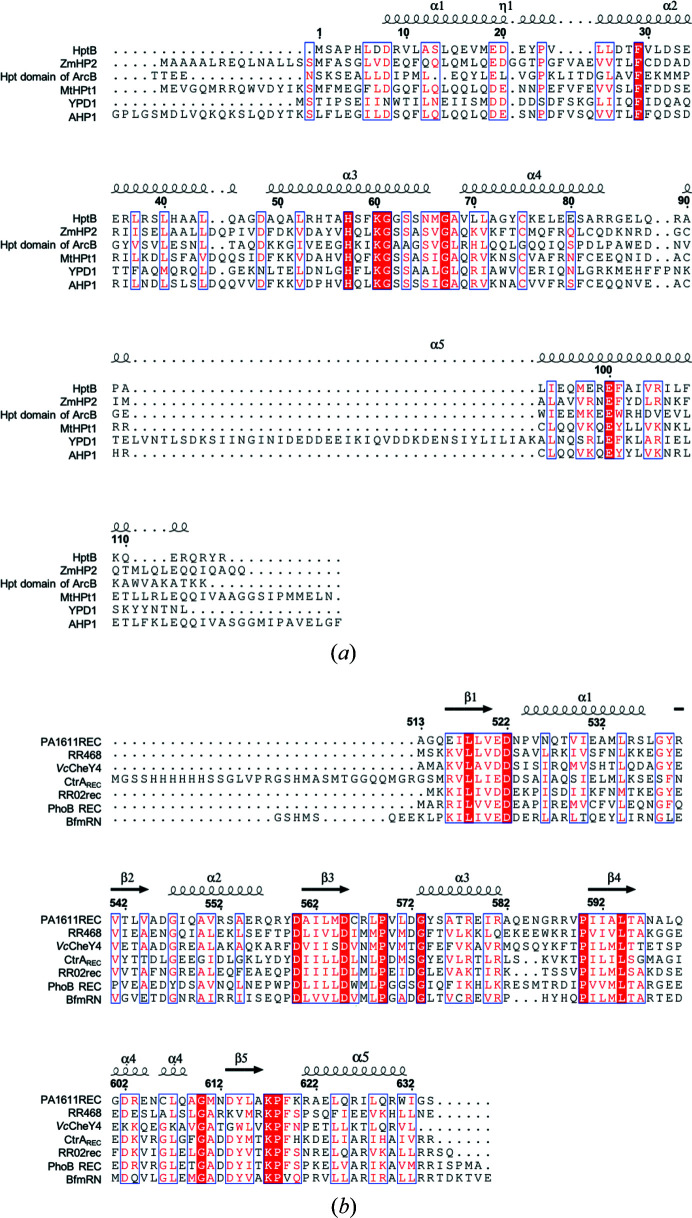
Information about secondary structures and sequence alignment of (*a*) HptB and (*b*) PA1611REC with structural homologues obtained using the *DALI* server. The sequence comparison was performed with the *ESPript* web server (http://espript.ibcp.fr/ESPript/ESPript/). The residues with high similarity appear in blue boxes; the conserved residues are white shaded in red. The species and PDB codes of each protein sequence are as follows: ZmHP2, *Zea mays*, 1wn0 (Sugawara *et al.*, 2005 ▸); Hpt domain of ArcB, *Escherichia coli*, 2a0b (Kato *et al.*, 1999*b* ▸); MtHPt1, *Medicago truncatula*, 3us6 (Ruszkowski *et al.*, 2013 ▸); YPD1, *Saccharo­myces cerevisiae*, 2r25 (Zhao *et al.*, 2008 ▸); AHP1, *Arabidopsis thaliana*, 4euk (Bauer *et al.*, 2013 ▸); RR468, *Thermotoga maritima*, 3gl9 (Casino *et al.*, 2009 ▸); *Vc*CheY4, *Vibrio cholerae*, 4h60 (Biswas *et al.*, 2013 ▸); CtrA_REC_, *Brucella abortus*, 4qpj (Willet *et al.*, 2015 ▸); RR02rec, *Streptococcus pneumoniae*, 1nxw (Bent *et al.*, 2004 ▸); PhoB REC, *Escherichia coli*, 1b00 (Sola *et al.*, 1999 ▸); BfmRN, *Acinetobacter baumannii*, 6br7 (Draughn *et al.*, 2018 ▸).

**Figure 3:**
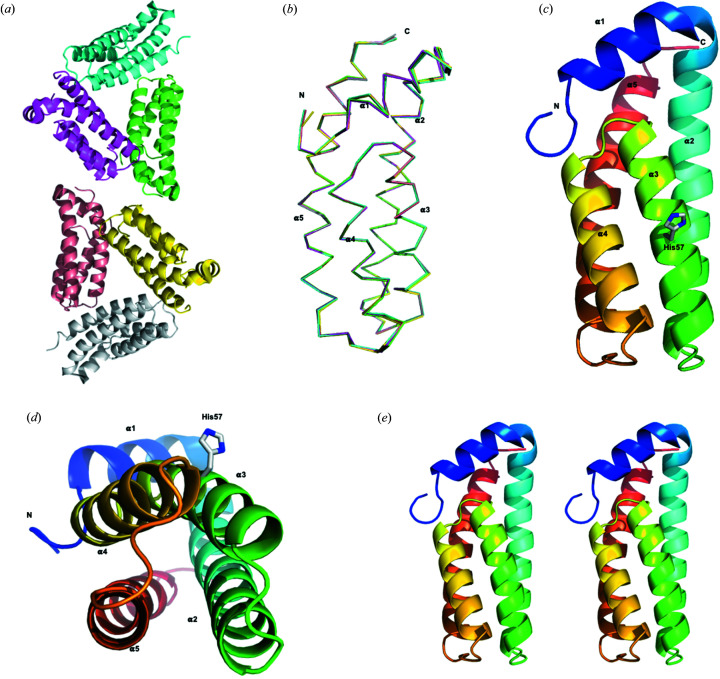
Overall structure of HptB. (*a*) Ribbon diagram illustrating the crystal packing in the native HptB crystal. There are six protein molecules in one asymmetric unit. (*b*) Diagram showing the structures are well superimposed among the six HptB protomers in the asymmetric unit. (*c*) Ribbon diagram illustrating the structural folding and secondary-structure elements of the HptB monomer. The helices are numbered sequentially α1 to α5 from the N-terminus to the C-terminus. The side-chain carbon and nitro­gen atoms of the active-site histidine (His57) are shown as white and blue sticks, respectively. (*d*) Bottom view of the structure from (*c*). The side chain of His57 is highly exposed to solvent. The four-helix bundle core is composed of helices α2, α3, α4 and α5. (*e*) Stereoview of the HptB structure. Helix α1 forms a cap on top of the four-helix bundle.

HptB consists of 116 amino acids with a total molecular mass of ∼13.2 kDa and shares a low-sequence identity with other Hpt proteins or domains [Fig. 2 ▸(*a*)]. Initially, we found crystallizing native HptB to be difficult. We suspected that Cys75 would introduce an intermolecular di­sulfide bond and interrupt the regular orientation of the protein in solution. We thus prepared SeMet-labeled mutant HptB–C75A for crystallization and phase determination. The crystals of SeMet-labeled mutant HptB–C75A belong to the tetragonal space group *I*4_1_22 with three protein molecules in one asymmetric unit. Then, we successfully crystallized the native HptB in another tetragonal space group, *P*4_3_2_1_2, with six protein molecules in one asymmetric unit. The two pairs of trimeric proteins related to the non-crystallographic twofold symmetry exhibit similar structures [Fig. 3 ▸(*a*)], but the protein is monomeric in solution, as confirmed by size-exclusion chromatography. The initial phases and structure of SeMet-labeled mutant HptB–C75A were first determined with the Se-MAD method at ∼2 Å. The crystal structure of native HptB was subsequently determined with the mutant HptB structure as an initial model and refined to high resolution (1.58 Å). The structural differences between mutant and native HptB are small, with an RMSD of 0.15 Å. The structures of six native HptBs in one asymmetric unit are essentially the same, with a small RMSD of 0.16 Å overall for the Cα atoms [Fig. 3 ▸(*b*)]. The final atomic model of native HptB comprises 115 residues, except for the first me­thio­nine. The entire main chain and most side chains are well defined with clear electron density.

**Figure 4:**
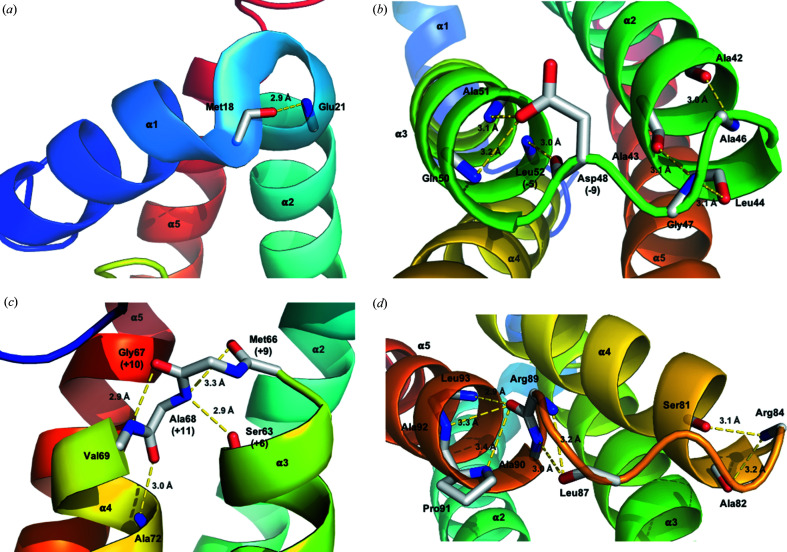
Hydrogen-bond linkages in the inter-helical regions of HptB. The diagrams illustrate the hydrogen bonds (yellow dashed lines) in the inter-helical regions between (*a*) helices α1 and α2; (*b*) helices α2 and α3; (*c*) helices α3 and α4; (*d*) helices α4 and α5. Carbon, nitro­gen and oxygen atoms of the residues involved in the hydrogen-bond linkages are shown as white, blue and red sticks, respectively. Most interactions occur through main-chain atoms of residues. With the exception of Asp48 (at position −9 from the active-site histidine His57) and Pro91, the side chains of residues have been omitted for clarity of the main-chain interactions. The distances listed are the corresponding values averaged over the six molecules in the asymmetric unit. The conserved residues involved in the hydrogen-bond linkages are labeled with numbers in parentheses to indicate their positions from the active-site histidine His57.

The structure of HptB comprises five α-helices and folds into an elongated α-helical bundle with an up-and-down topology. The helices α2 (Tyr22–Gln45), α3 (Ala49–Asn65), α4 (Val69–Arg83) and α5 (Ala90–Arg112) are twisted around the central axis and form an antiparallel four-helix bundle. Residues Ala46–Asp48, Met66–Ala68 and Arg84–Arg89 form short turns between helices α2–α3, α3–α4 and α4–α5, respectively. The four-helix bundle is covered with the short N-terminal helix α1 (Asp8–Met18) which protrudes into the solvent environment and connects to helix α2 with a 3_10_ helix (Glu19–Glu21) [Fig. 3 ▸(*c*)]. All these inter-helical regions are well ordered with some residues contributing to the stabilization of the helix-bundle through hydrogen bonds (Fig. 4 ▸).

The four-helix bundle is stabilized predominately by inter-helix hydro­phobic contacts contributed by the side chains of residues Ala3, Pro4 and Leu6 at the N-terminal loop; Leu11, Leu14, Val17 and Met18 at helix α1; Tyr22, Pro23, Val24, Leu25, Leu26, Phe29, Val30, Leu37, Leu40, Ala43 and Leu44 at helix α2; Ala49, Ala51, Leu52, Ala56 and Phe59 at helix α3; Met66 and Ala68 at the turn between helices α3 and α4; Leu70, Leu71, Tyr74, Leu78 and Ala82 at helix α4; Leu87 at the turn between helices α4 and α5; Ala90, Pro91, Ile94, Met97, Phe101, Ile103, Val104, Ile106, Leu107 and Phe108 at helix α5; and Tyr115 at the C-terminal loop. Helix α1 sits on top of the bundle to form a cap and provides additional stabilization [Fig. 3 ▸(*c*)]. Aside from directly contributing the hydro­phobic side chains to the inter-helix hydro­phobic contact, helix α1 helps to stabilize the bundle with the side chains of non-hydro­phobic residues, which are highly accessible on the surface. The non-hydro­phobic residues on helix α1 shield the hydro­phobic regions of helices α2 and α5, which are not covered by helices α3 and α4, and the hydro­phobic turn connecting helices α3 and α4 from exposure to solvent.

### 3.2. The conserved Hpt fold and structural features in the active site   

**Figure 5:**
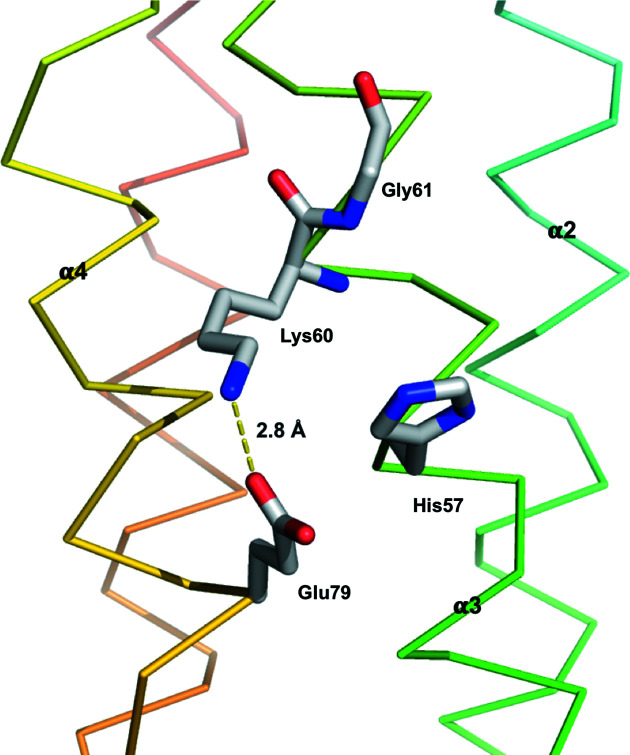
Structural features around the active-site histidine (His57) of HptB. The diagram shows the hydrogen-bond linkage (yellow dashed line) between Lys60 and Glu79 near the active-site histidine (His57) and the spatial position of the highly conserved glycine residue (Gly61) at position +4 from His57. The indicated distance is a value averaged over six molecules in the asymmetric unit. Carbon, nitro­gen and oxygen atoms of His57, Lys60, Gly61 and Glu79 are shown as white, blue and red sticks, respectively.

The protein family of Hpt proteins and domains could be further divided into enzymatically active and inactive orthologs. The active orthologs have a conserved histidine residue that serves as the phospho­relay active site (Ruszkowski *et al.*, 2013 ▸). In HptB, this conserved histidine residue, His57, is located near the middle of helix α3. The imidazole side chain of His57 protrudes from the bundle and is exposed to solvent [Figs. 3 ▸(*c*) and 3 ▸(*d*)]. The surrounding residues are featured so as to maximize the accessibility of His57. The hydrogen-bonding linkages between Lys60 and Glu79 prohibit their side chains from shielding the imidazole ring of the active-site histidine. High solvent accessibility of the imidazole ring is achieved through the nearby small side-chain volume, which results from the highly conserved glycine residue (Gly61 in HptB) at position +4 from the active-site histidine (Fig. 5 ▸).

Some conserved residues are related to the stabilization of the structural integrity. Aside from several residues contributed to inter-helix hydro­phobic contacts, the residues at positions −5 (Leu52 in HptB), −9 (Asp48), +6 (Ser63), +9 (Met66), +10 (Gly67) and +11 (Ala68) from the active-site histidine are conserved among Hpt proteins/domains with the structures determined listed in Fig. 2 ▸(*a*). The residues at positions −5 and −9 are involved in the hydrogen-bonding linkage between the first helix (α2 in HptB) and the second helix (α3), whereas the residues at positions +9, +10 and +11 are involved in the hydrogen-bonding linkage between the second helix (α3) and third helix (α4) of the bundle in the inter-helical region [Figs. 4 ▸(*b*) and 4 ▸(*c*)]. The hydrogen-bonding linkages include the hydrogen bonds between (i) the main-chain oxygen of Asp48 at position −9 and the main-chain nitro­gen of Leu52 at position −5 [Fig. 4 ▸(*b*)]; (ii) the side-chain oxygen of Asp48 at position −9 and the main-chain nitro­gen of Ala51 and Gln50 at positions −6 and −7, respectively [Fig. 4 ▸(*b*)]; (iii) the main-chain oxygen of Ser63 at position +6 and the main-chain nitro­gen of Ala68 at position +11 [Fig. 4 ▸(*c*)]; (iv) the main-chain oxygen of Met66 at position +9 and the main-chain nitro­gen of Ala68 at position +11 [Fig. 4 ▸(*c*)]; (v) the main-chain oxygen of Gly67 at position +10 and the main-chain nitro­gen of Val69 at position +12 [Fig. 4 ▸(*c*)]; (vi) the main-chain oxygen of Ala68 at position +11 and the main-chain nitro­gen of Ala72 at position +15 [Fig. 4 ▸(*c*)]. The N and C termini of the second helix (α3), at which the active-site histidine is located, are additionally stabilized due to these hydrogen bonds contributed by the conserved residues.

### 3.3. Overall structure of PA1611REC   

The recombinant PA1611REC protein comprises amino acids 507–651 of hybrid sensor histidine kinase PA1611 and contains the C-terminal receiver domain. The molecular mass of recombinant PA1611REC with a 6×His fusion tag is ∼18 kDa. The crystals of PA1611REC with Mg^2+^ belong to trigonal space group *P*3_1_21 with one protein molecule in the asymmetric unit. There are 122 amino-acid residues (a.a. 513–634) defined in the final atomic model; the electron-density map is clear enough to trace most parts of the main chains and side chains, except residues 507–512 and 635–651 were not seen, likely because of flexibility. The divalent magnesium ion (Mg^2+^) from the crystallization solution was found to be coordinated in the active-site cleft.

**Figure 6:**
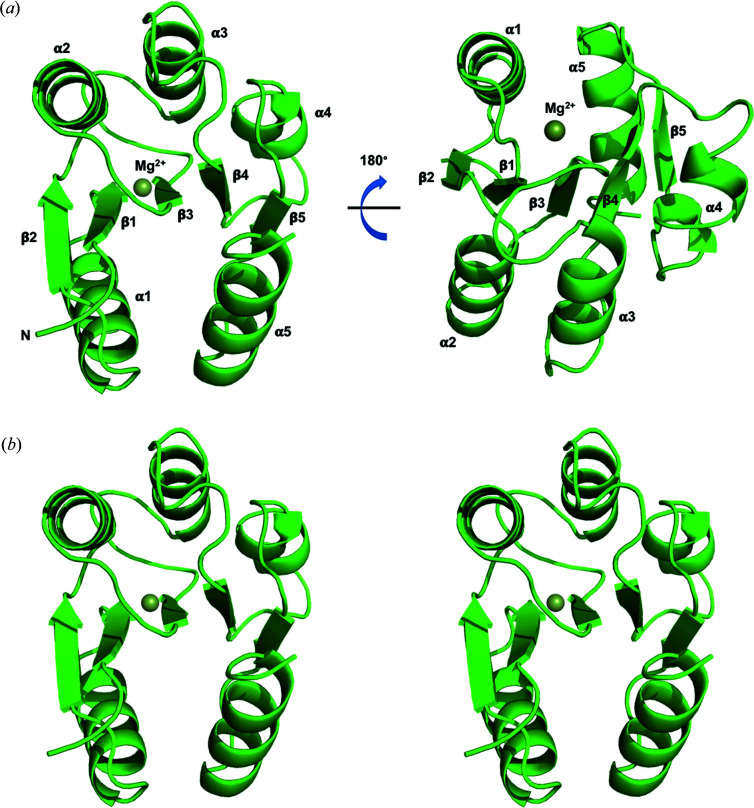
Overall structure of PA1611REC. (*a*) Ribbon diagram (green) illustrating the secondary-structure elements in PA1611REC. The β-strands and α-helices are numbered sequentially β1 to β5 and α1 to α5, respectively, from the N- to the C-terminus. Mg^2+^ is shown as a light green sphere. (*b*) Stereoview of the overall structure of PA1611REC with Mg^2+^ in the active-site cleft (PA1611REC-Mg^2+^).

The overall structure of PA1611REC possesses a conventional (β/α)_5_ topology as observed in other receiver domains of response regulators despite the fact they share low-sequence identities [Fig. 2 ▸(*b*)]. In this (β/α)_5_ motif, the five-stranded parallel β-sheets, which are β1 (Glu516–Val520), β2 (Arg540–Val544), β3 (Ala561–Asp565), β4 (Ile591–Thr595) and β5 (Asp613–Ala616), are folded in the central core. The core comprises β1, β3 and β4 which contain conserved hydro­phobic residues. The surrounding five α-helices are α1 (Pro524–Ser536), α2 (Gly547–Arg556), α3 (Gly573–Arg581), α4 (Gly601–Gln608) and α5 (Arg621–Trp631), among which two (helices α1 and α5) are located on one side, whereas the others (helices α2, α3 and α4) are on the opposite side. The β-strands and α-helices are connected with loops. Based on the assignment of the secondary structure from the *DSSP* web server, helix α4 is composed of two 3_10_ helices (Gly601–Glu604 and Cys606–Gln608) and separated at Asn605 (Fig. 6 ▸). An inspection of the secondary structural geometry shows that the φ and ψ angles of Asn605 are −150.3 and −100.1°, respectively, which deviate from the normal range for a helical structure. Moreover, the adjacent residue Cys606 is positioned structurally closely to Cys566 (on the loop β3→α3) to form a di­sulfide bridge. Interestingly, both Cys606 and Cys566 are unique in P1611REC. Two 3_10_ helices separated at Asn605 in helix α4 might be presumably affected by the di­sulfide formation of Cys606–566. This distinctive structural feature in PA1611REC, which does not exist in other receiver domains of response regulators, might contribute to the interaction with HptB through the residue on loop β4→α4 discussed later.

### 3.4. Metal binding in the active-site cleft of PA1611REC   

**Figure 7:**
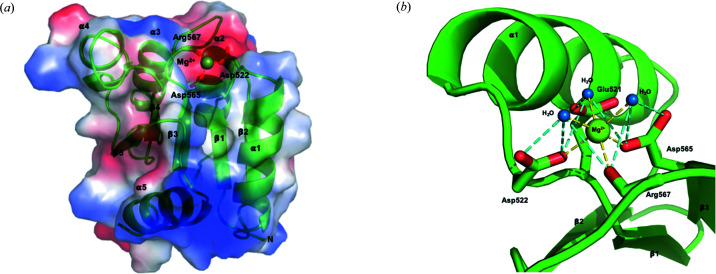
Binding of a divalent metal ion in the active-site cleft of PA1611REC. (*a*) Diagram of the surface electrostatic potential of PA1611REC. Negatively charged regions are colored red and positively charged regions blue, respectively. Divalent metal ion Mg^2+^ (shown as a light green sphere) is located in the negatively charged active-site cleft and interacts with Asp522, Asp565 and Arg567 (shown as sticks). (*b*) Spatial position of the residues and water molecules involved in the interactions with Mg^2+^. The six coordination positions of Mg^2+^ are indicated with yellow dashed lines, whereas the interactions between the residues and water molecules are indicated with cyan dashed lines. Oxygen atoms of Glu521, Asp522, Arg567 and Asp565 are shown as red sticks; water molecules are displayed as small blue spheres.

In PA1611REC, the conserved aspartate residue, Asp565, responsible for phospho­ryl group binding is located in the active-site cleft formed by loops β1→α1 (Glu521 to Asn523), β2→α2 (Ala545 to Asp546) and β3→α3 (Cys566 to Asp572) [Fig. 7 ▸(*a*)]. The hydrogen bond between the carbonyl oxygen of Cys566 and the amide nitro­gen of Gly573 on helix α3 contributes to the stabilization of the active-site cleft. Previous studies have shown that the binding of divalent metal ions in the active-site cleft is essential to activate the receiver domain (Stock *et al.*, 1993 ▸). The surface electrostatic potential around the active-site cleft of PA1611REC is highly negatively charged and suitable to accommodate metal ions [Fig. 7 ▸(*a*)]. The divalent magnesium ion (Mg^2+^) is hexa-coordinated, of which three positions coordinate with three water molecules, and three with the main-chain oxygen atom of Arg567 and the side-chain oxygen atoms of Asp522 and Asp565. The three water molecules coordinated to the magnesium ion form further hydrogen bonds with the main-chain oxygen atom of Arg567 and the side-chain oxygen atoms of Asp522 and Asp565. Moreover, one of these water molecules also interacts with Glu521 via hydrogen bonding [Fig. 7 ▸(*b*)].

### 3.5. The docking model of the HptB–PA1611REC complex   

The key point in phospho­relay is the recognition between Hpt proteins/domains and receiver domains of HK or RR. The complex structures of SLN1-R1/YPD1 (PDB entry 2r25; Zhao *et al.*, 2008 ▸) and AHK5_RD_/AHP1 (PDB entry 4euk) have highlighted that there is a general shape complementarity between the kidney-shaped Hpt protein/domain and the convex surface of the receiver domain (Zhao *et al.*, 2008 ▸; Bauer *et al.*, 2013 ▸). The transfer of the phospho­ryl group between PA1611 and HptB was confirmed with an *in vitro* phospho­relay assay using [γ-32P]ATP in our previous work (Lin *et al.*, 2006 ▸; Hsu *et al.*, 2008 ▸); however, our attempts to obtain the crystal structure of HptB in complex with the receiver domain of PA1611 were unsuccessful. We thus modeled the HptB–PA1611REC complex with the *HADDOCK* web server to elucidate the interactions between HptB and the cognate receiver domain. Previous studies have shown that the divalent metal ion is involved in the phospho­transfer reactions of the TCS transduction pathway; the relevant ion *in vivo* is presumably Mg^2+^ (Stock *et al.*, 2000 ▸). Divalent Mg^2+^ in the structure of PA1611REC was hence taken into consideration in the docking process. Moreover, as mentioned earlier, the structures of six HptBs in one asymmetric unit are essentially the same; thus, we selected one representative protomer for docking with PA1611REC.

**Figure 8:**
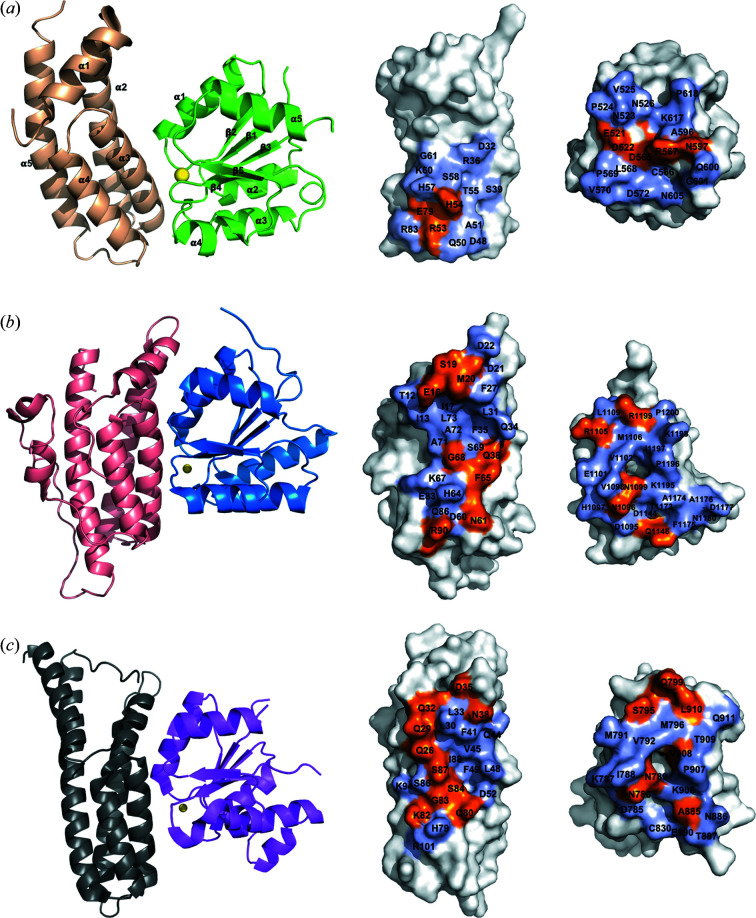
Overall structure and interface of (*a*) the HptB–PA1611REC complex model and the complexes of (*b*) SLN1-R1/YPD1 and (*c*) AHK5_RD_/AHP1. The ribbon diagrams illustrate (*a*) the docking model of the HptB–PA1611REC complex and the structures determined of (*b*) SLN1-R1/YPD1 and (*c*) AHK5_RD_/AHP1 complexes. The molecules of HptB, PA1611REC-Mg^2+^, SLN1-R1, YPD1, AHK5_RD_ and AHP1 are colored wheat, green, blue, salmon, magenta and dark gray, respectively. The Mg^2+^ ion is shown as a yellow sphere. The interface of the complexes, analyzed using the *PDBePISA* web server, is shown in the surface presentation. The residues involved in the formation of hydrogen bonds or salt bridges are colored orange, whereas the residues contributing to the hydro­phobic and van der Waals interactions are colored purple.

**Figure 9:**
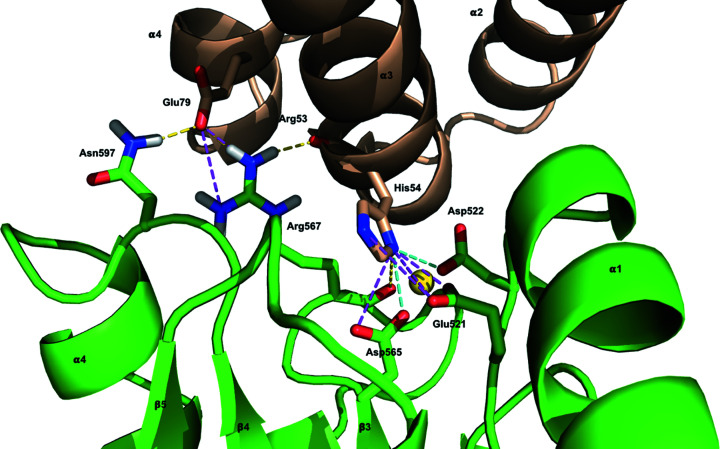
Interactions at the interface of the HptB–PA1611REC complex model. The molecules of HptB and PA1611REC-Mg^2+^ are colored wheat and green, respectively. The Mg^2+^ ion is shown as a yellow sphere. The main chains and side chains of the residues involved in the interface of the complex model analyzed with *PDBePISA* are depicted as sticks. Hydrogen bonds and salt-bridge interactions are represented as yellow dashed lines and magenta dashed lines, respectively. The interaction with both hydrogen bonds and salt-bridges are indicated with cyan dashed lines. Nitro­gen and oxygen atoms are depicted as blue and red sticks, respectively, and carbon atoms are the same color as the protein molecule.

According to the docking model, HptB and PA1611 REC would form a complex in a 1:1 ratio. Most of the interaction surface is composed of the second helix (helix α3) of the four-helix bundle of HptB, the N-terminus of helix α1 of PA1611REC, and the loops (β1→α1, β3→α3 and β4→α4) around the active-site cleft of PA1611REC [Fig. 8 ▸(*a*)]. According to an analysis calculated with the *PDBePISA* web server, there are 14 hydrogen bonds and salt bridges at the interface, in which 15 residues in HptB and 20 residues in PA1611REC are involved (Fig. 9 ▸, Table 3 ▸). Among the above 20 residues, the active-site residue Asp565 of PA1611REC also participates in the hydrogen-bonding network between HptB and PA1611REC. Although the active-site residue His57 of HptB is in the interface of the complex model, it is, however, not involved in the hydrogen bonds or salt bridges between the two proteins [Figs. 8 ▸(*a*) and 9 ▸]. There are few structural differences between the proteins of the *apo* form and the HptB–PA1611REC complex model, with an RMSD of 0.44 Å for HptB and 0.43 Å for PA1611REC. Furthermore, an inspection of of the residues of HptB involved in the interface of the complex model revealed that their side chains are positioned in consistent and similar orientations among the six HptB protomers in one asymmetric unit, suggesting that the docking model serves the representative complex.

## 4. Discussion   

### 4.1. Comparison of HptB with other Hpt proteins/domains   

**Figure 10:**
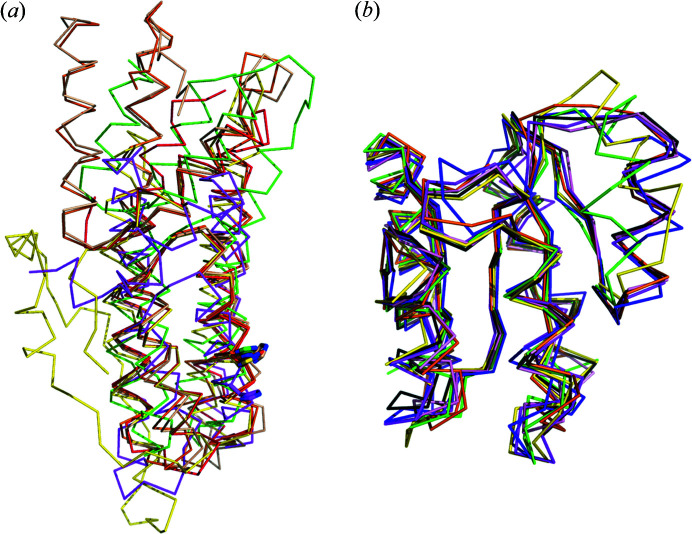
Structure alignment of (*a*) Hpt proteins/domains and (*b*) receiver domains listed in Fig. 2 ▸. (*a*) Overall structure of HptB, ZmHP2 and Hpt domain of ArcB, MtHPt1, YPD1 and AHP1 colored red, green, magenta, orange, yellow and wheat, respectively. The common structural motif, the four-helix bundle, is superimposed among these Hpt proteins/domains. Nitro­gen atoms of the active-site histidine are shown as blue sticks; the carbon atoms of the residues are shown as sticks in the same color as the protein. (*b*) Overall structure of PA1611REC, RR468, *Vc*CheY4, CtrA_REC_, RR02rec, PhoB REC and BfmRN colored green, magenta, purple blue, orange, dark gray, yellow and pink, respectively. Among these receiver domains, the main (β/α)_5_ architecture is well superimposed, except helix α4 which is is variable, especially helix α4 of PA1611REC.

HptB exhibits a low level of overall sequence similarity with other Hpt proteins/domains [Fig. 2 ▸(*a*)], but they all share a common structural feature as an elongated four-helix bundle, which is kidney-shaped with up-and-down topology and stabilized by inter-helix hydro­phobic contacts and hydrogen-bond linkages in the inter-helical regions. The conserved residues are located mainly at the helices forming the four-helix bundle and surrounding the active-site histidine (His57 in HptB), indicating that they contribute to the stability of the bundle and maximize the accessibility of the active-site histidine [Fig. 2 ▸(*a*)]. The active-site histidine is located at a similar position, which is near the middle of the second helix (α3 in HptB) of the helix bundle. The major difference is located at the N-terminal helix (α1 in HptB) and C-terminus beyond the four-helix bundle [Fig. 10 ▸(*a*)]. The differences in the segments outside the four-helix bundle might influence the specific interactions between separate Hpt proteins/domains in each species and the cognate receiver domains.

A search for structural homologs with the *DALI* server (Holm, 2019 ▸) showed that HptB bears structural similarity not only to other Hpt proteins/domains but also to proteins without functional similarities, such as flagellin (PDB entry 5maw; Altegoer, *et al.*, 2018 ▸) with a *Z*-score of 9.7, mannose-6-phosphate receptor binding protein 1 (PDB entry 1szi; Hickenbottom *et al.*, 2004 ▸) with a *Z*-score of 8.7 and cytochrome c′ (PDB entry 5b3i; Fujii *et al.*, 2017 ▸) with a *Z*-score of 8.5. The four-helix bundle motif is evidently suitable for diverse biological functions (Xu & West, 1999 ▸).

The orientation of helix α1 in HptB is similar to that of helix α2 in AHP1 of *Arabidopsis*. Helix α2 of AHP1 contributes additional hydrogen bonds and hydro­phobic interactions to the interface with the receiver domain of AHK5 (AHK5_RD_). Compared with the AHK5_RD_–AHP1 complex, the CheA_3_HP1 of *R. sphaeroides*, which lacks extra helices outside the conserved Hpt bundle, has shown a smaller contact area and affinity with its partner, CheY_6_. The difference in affinity indicates that the extra helix at the N-terminus of the four-helix bundle might play an important role in the interaction with the cognate receiver domains (Bauer *et al.*, 2013 ▸). Our docking model showed, however, that helix α1 of HptB is not involved in the interaction with the receiver domain of PA1611. The major role of this helix might therefore be to stabilize the four-helix bundle of HptB or to interact with other subjects, such as another domain of PA1611 or other proteins.

### 4.2. Structural features of HptB facilitates its interaction with receiver domains   

The hydrogen-bond linkages around the side chains of the residues near the active-site histidine have been suggested to facilitate its accessibility to the phospho­ryl group and to increase the nucleophilicity of the nitro­gen atom at the imidazole ring to improve the efficiency of phospho­transfer (Xu & West, 1999 ▸). Moreover, the glycine residue (Gly61 in HptB) at position +4 from the active-site histidine (His57 in HptB) is located on its adjacent ridge in the second helix of the bundle (α3 in HptB) and seems to carve a space for the histidine to expose its imidazole side chain to solvent. It has been suggested that, if an amino acid with a larger side chain replaces glycine, the activity of the active-site histidine to accept and to transfer a phospho­ryl group would be disturbed (Kato *et al.*, 1997 ▸). In YPD1 and the Hpt domain of ArcB, the phospho­rylation efficiencies were decreased when this glycine was replaced by Gln or Asp (Sugawara *et al.*, 2005 ▸). Aside from the active-site histidine and the glycine residue at position +4, the lysine residue at position +3 (Lys60 in HptB) is conserved throughout all active Hpt orthologs. The conservation and position imply that this lysine residue is involved in the phospho­relay process. The phospho­rylation efficiency of a mutant in which the +3 lysine was substituted with alanine was decreased in YPD1 (Janiak-Spens & West, 2000 ▸). In HptB, Lys60 contributes to the formation of the positively charged region around the active-site histidine, which may help to neutralize the negatively charged phospho­ryl group as well as form hydrogen bonds to the oxygen atoms of phospho­rylated histidine, providing an additional stabilizing force.

Although the sequence identity is low among Hpt proteins/domains, all Hpt proteins/domains share a common structural feature and active site, with some conserved residues that could be important for the structure and function described above. The key structural feature for the interactions between Hpt proteins/domains and receiver domains has been proposed to be the general shape complementarity between them, observed in several TCSs (Kato *et al.*, 1997 ▸; Zhao *et al.*, 2008 ▸; Bauer *et al.*, 2013 ▸). In contrast, non-conserved residues might be important for the specific recognition of molecular partners (Xu & West, 1999 ▸; Xu *et al.*, 2009 ▸). Although some Hpt proteins/domains could interact with non-cognate partners *in vitro* and *in vivo*, the rates of phospho­transfer in these cases might be substantially less than with cognate partners (Rogov *et al.*, 2004 ▸; Xu *et al.*, 2009 ▸). Because a different arrangement of amino acid residues would alter the affinity and specificity of the interaction between each Hpt and receiver domain, one reasonably suspects that the presence of Hpt proteins/domains could help to diminish or prevent the cross-interaction between distinct pathways in a TCS (Sugawara *et al.*, 2005 ▸; Xu *et al.*, 2009 ▸).

Aside from the general shape matching, other structural features might be involved in the interaction between Hpt proteins/domains and receiver domains. For example, some Hpt proteins/domains have a hydro­phobic cavity in the proximity of the active-site histidine. The hydro­phobic interaction has been proposed to contribute to the formation of the Hpt–REC complex; mutation of the hydro­phobic residues in that cavity would decrease the phospho­transfer activity (Kato *et al.*, 1999*a* ▸; Rogov *et al.*, 2004 ▸). The active-site histidine (His57) of HptB is surrounded largely by positively charged residues. We hence suggest that the electrostatic interactions and hydrogen bonding also help the binding of HptB and its partner *in vivo*. Based on an analysis of the complex model with the *PDBePISA* web server, the residues involved in the interface between HptB and PA1611REC contribute to the hydrogen bonds and salt bridges as well as hydro­phobic and van der Waals interactions [Figs. 8 ▸(*a*) and 9 ▸, Table 3 ▸].

### 4.3. Comparison of PA1611REC with other receiver domains   

The receiver domains typically exist in response regulators and also in some sensor histidine kinases in the hybrid TCS. Although the sequence similarity is not high, the receiver domain of PA1611 shares structural and functional homology with most bacterial receiver domains [Figs. 2 ▸(*b*) and 10 ▸(*b*)] (Lin *et al.*, 2006 ▸; Hsu *et al.*, 2008 ▸). The structure alignment of the receiver domains listed in Fig. 2 ▸(*b*) shows that the secondary structures are organized in a similar pattern with a typical (β/α)_5_ topology [Fig. 10 ▸(*b*)]. The central β strands and the regions around the active-site cleft are highly conserved [Fig. 2 ▸(*b*)]. In contrast, the region containing helix α4 and the loop β4→α4 displays a large variability, which causes an overall RMSD above 0.6 Å between PA1611REC and other receiver domains listed in Fig. 2 ▸(*b*). The RMSD values between PA1611REC-Mg^2+^ and RR468, *Vc*CheY4, CtrA_REC_, RR02rec, PhoB REC and BfmRN are 0.92, 0.95, 0.67, 0.79, 0.78 and 0.98 Å, respectively.

The conserved residues of the receiver domains are located mainly on β-strands and α-helices. Aside from the aspartate residue that is directly phospho­rylated (Asp565 in PA1611REC), highly conserved residues are involved in the process of phospho­relay. The aspartate residue at the end of β1 (Asp522 in PA1611REC) participates in the binding of the divalent metal ion. The structure of the CheY receiver domain with beryllofluoride (phospho­ryl group analog) in the active-site cleft (PDB entry 1fqw; Lee *et al.*, 2001 ▸) shows that the ligand is located close to the metal ion and is stabilized by Mg^2+^, conserved Thr87 on β4 with a hydrogen bond, and conserved Lys109 on β5 by salt-bridge interactions (Lee *et al.*, 2001 ▸). We hence suggest that Thr595 (Thr87 in CheY) and Lys617 (Lys109 in CheY) in PA1611REC would also interact with the phospho­ryl group and stabilize the conformation of the activated receiver domain during phospho­rylation. The alanine residue (Ala596 in PA1611REC) following Thr595 was thought to improve access of the conserved Thr/Ser to the phospho­rylation site (Bourret, 2010 ▸).

Although the α→β loops opposite the active-site cleft demonstrate greater variability in sequence and length, the β3→α3 loop near the active-site cleft is highly conserved. This loop has been suggested to form a universal recognition element across the receiver domain superfamily (Usher *et al.*, 1998 ▸). Moreover, an inspection of temperature *B*-factors of PA1611REC reveals that the β3→α3 loop and the other two loops (β1→α1, β2→α2) surrounding the active-site cleft are relatively rigid, compared with the loops away from the active site (see Fig. S1 of the supporting information). In our complex model, this β3→α3 loop participates in the interaction with HptB. We hence suggest that the β3→α3 loop in PA1611REC would be involved in the binding with HptB *in vivo*.

### 4.4. Structural alterations in receiver domains   

Conformational changes due to phospho­rylation have been assumed to be common in proteins related to signal transduction. For example, in *E. coli* CheY, a series of structural changes occur near the active-site cleft during phospho­rylation (Cho *et al.*, 2000 ▸; Lee *et al.*, 2001 ▸). The structures of *Thermotoga maritima* CheY (TMY) indicated that Ser82 and Phe101 might be sensitive to the phospho­rylation status of the active-site aspartate and alter the conformation simultaneously. In the unphospho­rylated state, the side chain of Phe101 is exposed to solvent; the side-chain hydroxyl of Ser82 points toward Phe101. After TMY is phospho­rylated, the side-chain hydroxyl of Ser82 is oriented towards the active site; the aromatic ring of Phe101 is buried (Usher *et al.*, 1998 ▸). Similar conformational changes have been reported for the residue substitutions in other receiver domains (Cho *et al.*, 2000 ▸; Lee *et al.*, 2001 ▸; Zhao *et al.*, 2008 ▸). In our determined structure of PA1611REC, there is no phosphate group in the active-site cleft. The side chain of Tyr614 (Phe101 in TMY) is exposed to solvent; the side chain of Thr595 (Ser82 in TMY) points towards it. The results suggest that our PA1611REC is in an inactivated form; there might be conformational changes that occur in the side chain of Thr595 and Tyr614 after phospho­rylation, similar to the residue substitutions in *E. coli* CheY and TMY.

Helix α4 and its adjacent regions display a great variability in several structures of RR receiver domains. In previous studies, these regions were shown to undergo conformational changes in the activated receiver domain (Cho *et al.*, 2000 ▸; Lee *et al.*, 2001 ▸; Zhao *et al.*, 2008 ▸). Aside from helix α4, the N-terminus of helix α1 has been reported to be partially unfolded when CheY interacts with cognate Hpt proteins/domains (Kato *et al.*, 1999*a* ▸). The conformational alterations of activated REC are varied. For example, in the YPD1/SLN1-R1 complex with Mg^2+^ and BeF_3_^−^, conformational changes of a significant number were observed in comparison with the *apo* complex. In contrast, there are very few conformational changes in the Spo0B/Spo0F-Mg^2+^-BeF_3_^−^ complex compared with the *apo* complex (Varughese *et al.*, 2006 ▸; Zhao *et al.*, 2008 ▸). Although the loops and some residues may undergo conformational changes during phospho­rylation, there is no dramatic structural rearrangement in the main secondary-structure architecture of receiver domains (Lee *et al.*, 2001 ▸). For example, the difference is small between the structures of BeF_3_^−^BfmRN and the *apo* form BfmRN, with an RMSD of 0.315 across 227 Cα atoms (Draughn *et al.*, 2018 ▸). The structural differences between PA1611REC in the *apo* form and the complex model are also small, with an RMSD of 0.43 Å.

### 4.5. Interactions among the receiver domain, metal ion and Hpt   

Previous studies have shown that a divalent metal cation helps to stabilize the phospho­rylated active-site aspartate residue of the receiver domain. In the study of EL_LovR, a protein with only one receiver domain in *Erythrobacter litoralis*, its lack of divalent metal ion was suggested to create electrostatic repulsion among the negatively charged residues around the active-site cleft which destabilize the protein (Ocasio *et al.*, 2015 ▸). The manners for metal binding are similar in most receiver domains, in which three coordination positions occur with the atoms of amino-acid residues and the others with solvent molecules. The structure of the CheY receiver domain with the phospho­ryl group analog, beryllofluoride, in the active-site cleft (PDB entry1fqw; Lee *et al.*, 2001 ▸) shows that beryllofluoride is located close to the metal ion; O_δ_ on the side chain of the conserved active-site aspartate (Asp57 in CheY) can interact with Mg^2+^ and the beryllium atom.

The studies of CheY have shown that Mn^2+^ has the same geometry as Mg^2+^ in the active-site and can also coordinate to Asp13, Asp57, Asn59 and BeF_3_^−^ (Lee *et al.*, 2001 ▸). The structure of PA1611REC with Ca^2+^ in the active-site cleft was also determined from a crystal grown in a crystallization solution containing Ca^2+^ (Fig. S2). The overall structures of PA1611REC-Mg^2+^ and PA1611REC-Ca^2+^ are similar with an RMSD of 0.23 Å. These results coincide with suggestions from previous studies that the divalent metal ion would not induce a conformational change of the REC active-site cleft, although it is required for the phospho­rylation process (Lee *et al.*, 2001 ▸).

The overall arrangement between Hpt proteins/domains and REC is similar in complex structures of SLN1-R1/YPD1, AHK5_RD_/AHP1 and our docking model of HptB–PA1611REC. However, compared with SLN1-R1/YPD1 and AHK5_RD_/AHP1 complexes, the slight deflection of the relative positions of HptB and PA1611REC results in fewer residues involved in the interface of our complex model according to an analysis with the *PDBePISA* web server (Fig. 8 ▸). The relative positions of the active-site residues in HptB (His57) and PA1611REC (Asp565) are also slightly different from those in SLN1-R1/YPD1 and AHK5_RD_/AHP1 complexes because of the minor deflection. We hence suggest the interactions between the two proteins in the HptB–PA1611REC complex model are weaker than those in SLN1-R1/YPD1 and AHK5_RD_/AHP1 complexes. Perhaps for this reason we could not directly observe the interaction between HptB and PA1611REC in the experiment using size-exclusion chromatography, and hence could not successfully obtain suitable crystals of the HptB–PA1611REC complex. Because the transfer of the phospho­ryl group between the cytosolic part of PA1611 (a.a. 202–652) and HptB has been confirmed with [γ-32P]ATP in our previous work (Lin *et al.*, 2006 ▸; Hsu *et al.*, 2008 ▸), we surmise that another part in addition to REC of PA1611 or some other molecule is necessary to enhance the interaction with HptB.

According to previous studies and an analysis with the *PDBePISA* web server, the active-site histidine residues of Hpt-related proteins in SLN1-R1/YPD1, AHK5_RD_/AHP1 and the HptB–PA1611REC model are involved in the interface of the complex but do not participate in the hydrogen-bond network and salt bridges of the binding surface (Figs. 8 ▸ and 9 ▸; Table 3 ▸). The conserved lysine residue at position +3 and glycine residue at position +4 from the active-site histidine also contribute to the interaction. These two residues in YPD1 and AHP1 are involved in the hydrogen-bond network or salt bridges of the interface, whereas the two residues (Lys60 and Gly61) in HptB are not involved in the hydrogen bonds and salt bridges between the two proteins in the complex model. Previous studies of the SLN1-R1/YPD1 complex described how the side chain of Gln86 in YPD1, which corresponds to Glu79 in HptB, forms a hydrogen bond with Gln1146 of SLN1-R1 (Xu *et al.*, 2003 ▸). An analysis of the interface in the HptB–PA1611REC complex model with the *PDBePISA* web server showed that Glu79 of HptB is also involved in the hydrogen-bond networks between the two proteins [Figs. 8 ▸(*a*) and 9 ▸; Table 3 ▸]. Glu79 of HptB might contribute to the interaction with the cognate receiver domain *in vivo*.

The aspartate residue (Asp565) responsible for phospho­ryl group binding in PA1611REC forms a hydrogen bond with His54 of HptB according to an analysis with the *PDBePISA* web server. In contrast, the conserved aspartate residue in AHK5_RD_ is not involved in the interface of the complex. Previous studies of the SLN1-R1/YPD1 complex described that this aspartate residue in SLN1-R1 does not participate in the interface of the complex, although our analysis with the *PDBePISA* web server shows that it is involved in the interaction between SLN1-R1 and YPD1. The N-terminus of helix α1 and the loop β1→α1, which is one of the loops around the active-site cleft, are highly conserved in structures and sequences among SLN1-R1, AHK5_RD_ and PA1611REC. These two segments contribute largely to the interface of the complex. Loop β3→α3 is structurally conserved and contributes largely to the interaction between HptB and PA1611REC, whereas in loop β3→α3 of SLN1-R1 and AHK5_RD_, only one non-conserved residue is involved in the binding surface of the complex. Loops β4→α4 of SLN1-R1, AHK5_RD_ and PA1611REC also majorly contribute to the interface of the three complexes, but these loops display a low level of sequence similarity. According to these analyses, we suggest that the loops around the active-site cleft of REC are not only involved in the interaction with Hpt proteins/domains but also might influence the specificity of recognition.

The properties and distributions of the amino-acid residues involved in the interactions vary across different complexes. The HptB–PA1611REC complex model, SLN1-R1/YPD1 and AHK5_RD_/AHP1 are all well conserved in the structures of their components and the overall organization of the complexes. However, the amino-acid residues contributing to hydrogen bonds, salt bridges and non-polar contacts are distributed differently across the interfaces (Fig. 8 ▸) (Zhao *et al.*, 2008 ▸; Bauer *et al.*, 2013 ▸).

## Supplementary Material

Supporting figures. DOI: 10.1107/S2052252520009665/lz5039sup1.pdf

PDB reference: PA1611REC-Mg^2+^, 7c1j

PDB reference: PA1611REC-Ca^2+^, 7cfw

PDB reference: WT-HptB, 7c1i

## Figures and Tables

**Table 1:** The DNA primers used in this study

Primers	Sequence (5′→3′)	Comment
HptB-C75A-Fw	CTCCTCGCCGGCTACGCGAAGGAGCTGGAGGAAAG	Mutation of HptB Cys75 to Ala
HptB-C75A-Rv	CTTTCCTCCAGCTCCTTCGCGTAGCCGGCGAGGAG	Mutation of HptB Cys75 to Ala
PA1611REC-Fw	CTAGCTAGCGACGCCGCCCCG	Cloning of PA1611REC into pET28a
PA1611REC-Rv	GGAATTCTCATTCCGGCTCCCC	Cloning of PA1611REC into pET28a

**Table 2:** Data collection and refinement statistics

	HptB-C75A (Se-peak)	HptB-C75A (Se-inflection)	HptB-C75A (Se-remote)	WT-HptB	PA1611 REC (Mg^2+^)	PA1611 REC (Ca^2+^)
PDB entry				7c1i	7c1j	7cfw
Data collection						
Beamline	BL13B1	BL13B1	BL13B1	BL15A1	BL15A1	BL15A1
Wavelength (Å)	0.9790	0.9792	0.9639	1.0000	1.0000	1.0000
Temperature (K)	110	110	110	110	110	110
Space group	*I*4_1_22	*I*4_1_22	*I*4_1_22	*P*4_3_2_1_2	*P*3_1_21	*P*3_1_21
Cell dimensions (Å)						
*a*	120.54	120.40	120.40	119.17	57.96	55.07
*b*	120.54	120.40	120.40	119.17	57.96	55.07
*c*	162.56	162.42	162.43	171.95	67.60	68.96
Resolution (Å)†	30–2.04 (2.11–2.04)	30–2.05 (2.12–2.05)	30–2.01 (2.08–2.01)	30–1.58 (1.64–1.58)	30–1.35 (1.40–1.35)	30–1.31 (1.36–1.31)
Completeness (%)†	92.3 (90.6)	93.4 (96.6)	93.3 (95.1)	98.4 (99.6)	97.8 (99.9)	94.0 (93.8)
Average redundancy	6.9	5.0	5.1	14.5	6.3	7.3
〈*I*/σ_*I*_〉†	15.3 (4.1)	14.5 (4.9)	14.5 (4.2)	36.3 (4.2)	18.4 (1.64)	16.0 (2.4)
*R*_sym_(%)† ‡	11.1 (39.7)	9.8 (28.7)	9.7 (30.8)	7.2 (76.1)	6.9 (125.5)	9.6 (103.6)
						
Refinement						
Resolution (Å)	–	–	–	30–1.58	30–1.35	30–1.31
*R*_work_ §/*R*_free_ ¶ (%)	–	–	–	19.3/22.5	18.0/21.6	17.6/21.3
No. of atoms						
Protein	–	–	–	6734	1110	1125
Heteroatom	–	–	–	–	1 (Mg^2+^)	1 (Ca^2+^)
Water	–	–	–	1230	173	222
Average *B* factors (Å^2^)						
Protein	–	–	–	22.91	27.56	17.09
Heteroatom	–	–	–	–	16.65	12.45
Water	–	–	–	43.11	43.66	35.04
R.m.s. deviations						
Bond lengths (Å)	–	–	–	0.013	0.013	0.015
Bond angles (°)	–	–	–	2. 06	2.02	1.84
						
Ramachandran plot						
Favored regions (%)	–	–	–	98.67	95.00	95.87
Allowed regions (%)	–	–	–	1.33	5.00	4.13
Outliers (%)	–	–	–	0	0	0

† Values in parentheses are for the highest-resolution shell.

‡ *R*_sym_ =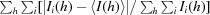 , where *I* *i* is the *i*th measurement and 〈*I*(*h*)〉 is the weighted mean of all measurements of *I*(*h*).

§ *R*_work_ = 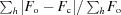, where *F*_o_ and *F*_c_ are the observed and calculated structure factor amplitudes of reflection *h*, respectively.

¶ *R*_free_ is the same as *R*_work_, but calculated with 5% of randomly chosen reflections omitted from refinement.

**Table 3:** *PDBePISA* analysis at the interface of the HptB–PA1611REC complex model

HptB residue (atom)			PA1611REC residue (atom)	
Hydrogen bonds				
His54 (ND1)	↔		Asp565 (OD2)	
His54 (ND1)	↔		Asp522 (OD1)	
His54 (ND1)	↔		Arg567 (O)	
Arg53 (O)	↔		Arg567 (HH21)	
Glu79 (OE1)	↔		Asn597 (HD21)	
Glu79 (OE1)	↔		Arg567 (HH22)	

Salt-bridge interactions				
His54 (ND1)	↔		Glu521 (OE1)	
His54 (ND1)	↔		Glu521 (OE2)	
His54 (ND1)	↔		Asp565 (OD1)	
His54 (ND1)	↔		Asp565 (OD2)	
His54 (ND1)	↔		Asp522 (OD1)	
His54 (NE2)	↔		Glu521 (OE1)	
Glu79 (OE1)	↔		Arg567 (NH1)	
Glu79 (OE1)	↔		Arg567 (NH2)	

Hydro­phobic and van der Waals interactions				
Asp32	↔		Val525	
Arg36	↔		Asn523	
Arg36	↔		Pro524	
Arg36	↔		Val525	
Ser39	↔		Pro524	
Asp48	↔		Pr0569	
Asp48	↔		Val570	
Gln50	↔		Cys566	
Gln50	↔		Leu568	
Gln50	↔		Pro569	
Gln50	↔		Asp572	
Gln50	↔		Asn605	
Ala51	↔		Pro569	
Thr55	↔		Asn523	
His57	↔		Ala596	
His57	↔		Lys617	
His57	↔		Pro618	
Ser58	↔		Asn523	
Ser58	↔		Asn526	
Lys60	↔		Lys617	
Gly61	↔		Pro618	
Arg83	↔		Gln600	
Arg83	↔		Gly601	
Arg83	↔		Asn605	
