# Supplementary material for: Structural insights into the histidine-containing phospho­transfer protein and receiver domain of sensor histidine kinase suggest a complex model in the two-component regulatory system in Pseudomonas aeruginosa
Source: IUCrJ. 2020 Aug 25;7(Pt 5):934–48. doi: 10.1107/S2052252520009665 (PMC7467158; doi:10.1107/S2052252520009665)
Supplement: Supplementary file 1 [file m-07-00934-sup1.pdf]

IUCrJ

Volume 7 (2020)

Supporting information for article:

Structural insights into the histidine-containing phosphotransfer protein and receiver domain of sensor histidine kinase suggest a complex model in the two-component regulatory system in *Pseudomonas aeruginosa*

Shao-Kang Chen, Hong-Hsiang Guan, Pei-Hsun Wu, Li-Ting Lin, Meng-Chun Wu, Hwan-You Chang, Nai-Chi Chen, Chien-Chih Lin, Phimonphan Chuankhayan, Yen-Chieh Huang, Pei-Ju Lin and Chun-Jung Chen

Supporting Information

For

Structural insights into histidine-containing phosphotransfer protein and receiver domain of sensor histidine kinase suggest a complex model in two-component regulatory system in *Pseudomonas aeruginosa*

Shao-Kang Chen[a,b], Hong-Hsiang Guan[b], Pei-Hsun Wu[b], Li-Ting Lin[b], Meng-Chun Wu[a,b], Hwan-You Chang[c], Nai-Chi Chen[b], Chien-Chih Lin[b], Phimonphan Chuankhayan[b], Yen-Chieh Huang[b], Pei-Ju Lin[b,d], Chun-Jung Chen[a,b,e,f*]

# of Supplementary Figure: 2

Supplementary Figure

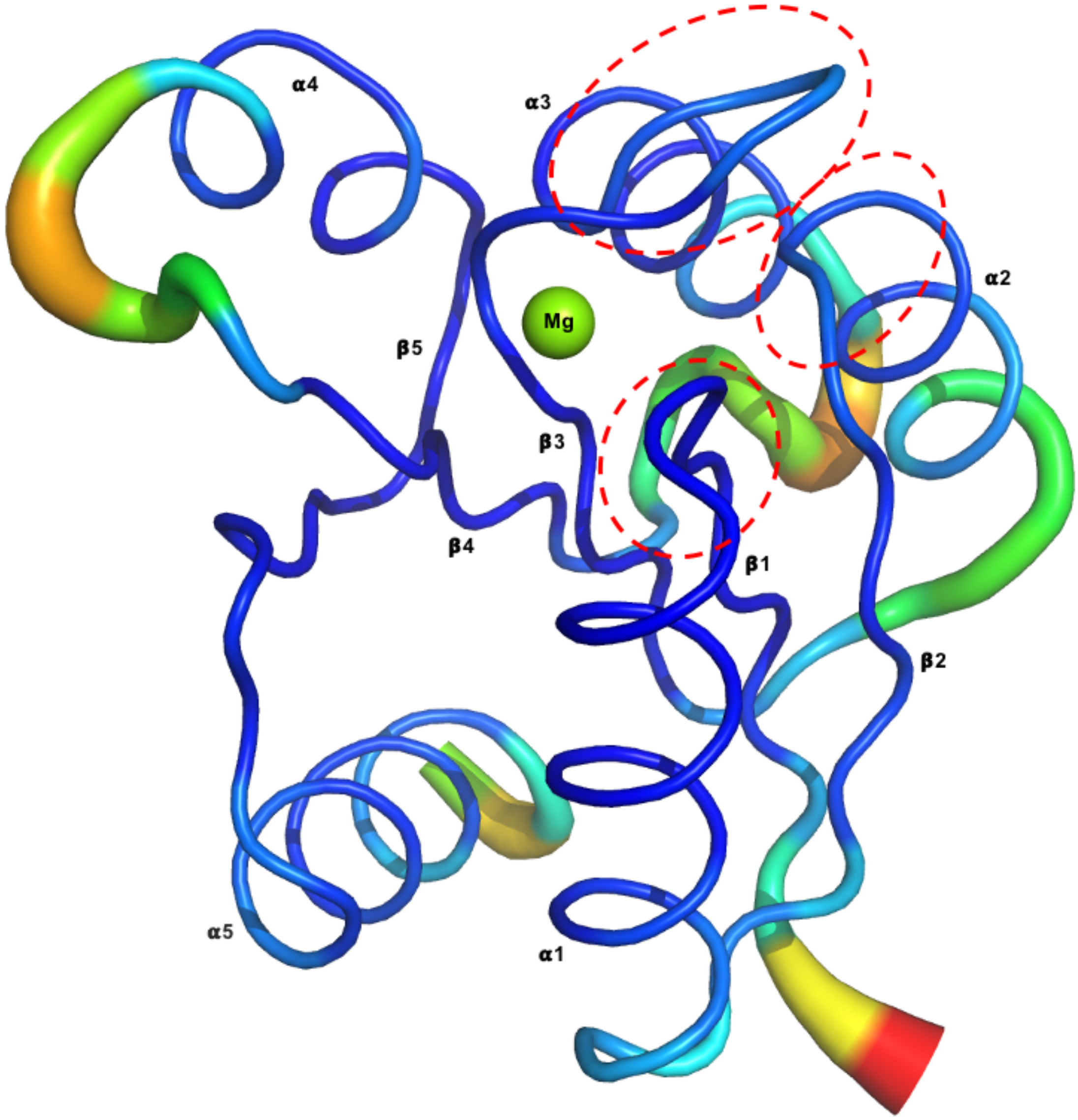

Figure S1. A temperature *B*-factor analysis of PA1611REC. The loops β1→α1 (Glu521 to Asn523), β2→α2 (Ala545 to Asp546) and β3→α3 (Cys566 to Asp572) surrounding the active-site cleft are relatively rigid with low B-factors compared to the loops away from the active site. Mg^{2+} is shown as a green sphere. *B*-factor decrement is presented from red to blue.

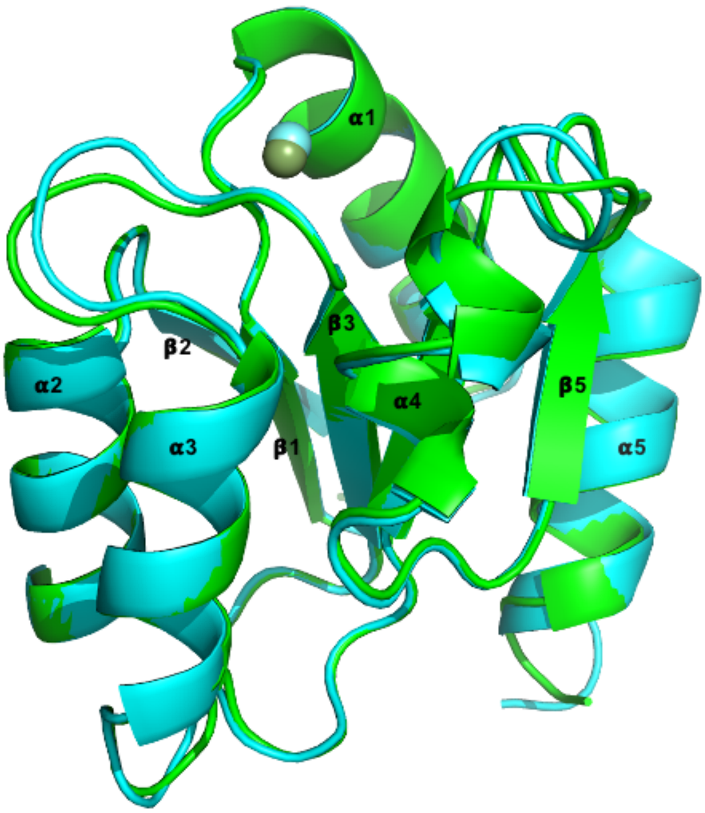

Figure S2. Structural comparison of PA1611REC-Mg^{2+} and PA1611REC-Ca^{2+}. The PA1611REC with Mg^{2+} in the active-site cleft is colored green, whereas the protein with Ca^{2+} in the active-site cleft is colored cyan. The β-strands and α-helices are numbered sequentially β1 to β5 and α1 to α5 from the N-terminus to the C-terminus. Mg^{2+} is shown as a yellow-green sphere, and Ca^{2+} is shown as a cyan sphere. The main $(\beta/\alpha)_5$ architecture is well superimposed, including the discontinuous helix α4.
